# Men living with HIV in serodiscordant relationships who desire a child/children

**DOI:** 10.7448/IAS.20.2.21749

**Published:** 2017-03-08

**Authors:** Raoul Fransen-dos Santos, Mauro Guarinieri

**Affiliations:** ^a^ International Civil Society Support, Amsterdam, The Netherlands; ^b^ The Global Fund to Fight AIDS, Tuberculosis and Malaria, Community, Rights & Gender Department, Geneva, Switzerland

**Keywords:** HIV, HAART, serodiscordant couples, pregnancy, stigma

Prior to the widespread use and availability of effective antiretroviral treatment [1], if you were living with HIV, having children was often not perceived to be an option – at least by many people living with HIV and often even more so by service providers [2]. Many of us living with HIV discarded a desire to have children from the moment of diagnosis – sometimes out of fear of transmission, sometimes based on the belief of having a limited life expectancy or for other reasons related to lack of knowledge and views in the early days of the epidemic. Even after the introduction of HAART, discussions about pregnancy, parenting and sex in general were often loaded with misinformation, stigma and judgment. Many people considered people living with HIV not to be fit for parenting, that having a baby was simply irresponsible, that having sex was selfish – and if sex was going to be a reality, HIV-positive people should only have sex with other people who were also HIV positive. Many people living with HIV were in agreement with this thinking which resulted in many of us, on diagnosis, foregoing the right and the dream to parent a biological child. Different kinds of parenting options were considered which also highlighted some of the policy challenges [3] still facing people living with HIV today.

For some people living with HIV, the desire whether or not to have children was not negatively influenced by their diagnosis. However, acting on this desire was frequently made difficult; not by the virus, but by the attitudes of friends and family, of healthcare providers and of faith – and spiritual leaders either out of worry for children growing up without their parent(s) or out of concern for preventing additional infections [4]. This was especially true for HIV-positive men in serodiscordant relationships. Making a truly informed decision was challenging. For many providers, but also peers, the importance of prevention overruled the rights aspects of these informed decisions. Public health reasoning or simple stigma (experienced and internal) had greater weight than principles of human rights and gender equity, making it especially challenging for men living with HIV to even consider accessing sexual and reproductive health services. And this is a challenge we have as yet not fully addressed. Only a few physicians provided support not based on stigma or personal views and these were often the leading HIV specialists in countries which exacerbated the divide for those living with HIV in more remote areas.

To a large extent, the fear of onward transmission, when it comes to the desire to have children, is experienced differently by men than women living with HIV in serodiscordant couples. For women, the fear would predominantly be related to concern about risk of infection to the child. For men, it would be about concern around risk of infecting their partner and for being blamed as the “source” of infection. However, how this fear manifests itself, the emotional struggle and how it impacts the decision-making on conceiving can be quite similar. Again, the support, or rather lack thereof, from healthcare professionals was and still can be instrumental in changing not only the perception of risk, but also in coming to a more informed decision, based on the right information and access to safe conception and pregnancy.

The onset of ART started to bring about change, not only to life expectancy, but also to attitudes of doctors and nurses in their support for safer conception as part of a comprehensive package of services. For many people living with HIV, this shift was instrumental in shaping and influencing their choices around having children, in particular for those not involved in advocacy, activism or working in communities, for whom an empowering message from their physician or frontline healthcare provider makes all the difference. This is particularly the case for men living with HIV, as guidance and early efforts on prevention of vertical transmission were largely focused on women living with HIV. The more recent pivot towards treatment as prevention with its strong scientific base [5] has galvanized a more robust universal approach and understanding which is more inclusive of men living with HIV.

An interesting perspective is that of younger men living with HIV (who may have been born with HIV) and who are growing up in an era where treatment as prevention is a reality and where PrEP is becoming more widely available for their sexual partners who are HIV negative. This is a significantly different environment from those who came of age in the days when HIV equalled AIDS and AIDS equalled death. The fear of a new, deadly disease made prevention campaign messaging define safer sex as condom use, which became standard practice for many people living with HIV. Successful efforts in reducing stigma and discrimination, including self-stigma, coupled with the scientific backing of being relatively unable to transmit the virus when on regular treatment, made it much easier to leave condoms behind and for men living with HIV to conceive without having to seek access to specialized and bespoke sexual and reproductive health services.

These achievements in the scientific and advocacy communities have been instrumental in shaping the attitudes and biases of many antenatal service providers in their support to men and women living with HIV, as well as in the personal environment – families and friends of women and men living with HIV. However, much is still to be done. Protocols for inclusive service provision have often not kept pace with the science. In many countries today, for example, it remains incredibly and unnecessarily challenging for couples where one or both partners are living with HIV to adopt a child or serve as a foster family. This stresses the continued importance of empowering messages and support from a range of health and social service providers which remains crucial to enable individuals and couples affected by HIV and especially serodiscordant couples to be supported in decision-making around both safer conception and also fostering and adoption options.

Increasingly men living with HIV are involved in serodiscordant relationships and their desire to fulfil their fatherhood aspirations is becoming a growing reality for many health and social services. Treatment access has become a game-changer for many, but societal attitudes and understanding of “living with HIV” in a serodiscordant relationship may take longer to change.
